# Management Strategies for Open Bite Relapse: A Systematic Review and Meta-Analysis

**DOI:** 10.7759/cureus.56285

**Published:** 2024-03-16

**Authors:** Mohammad K Alam, Afnan Alayyash

**Affiliations:** 1 Department of Preventive Dentistry, College of Dentistry, Jouf University, Sakaka, SAU

**Keywords:** open bite, open bite relapse, orthodontic treatment, orthodontic management, orthodontic appliance, orthodontic surgery, orthodontic retention

## Abstract

The purpose of orthodontic therapy is to correct malocclusion and produce a stable outcome that endures over time. Long-term stability can be difficult to achieve, and many patients relapse after treatment, particularly in instances of open bite relapse (OBR). This systematic review aimed to analyze different types of management strategies for OBR and conduct a meta-analysis to find the best method of dealing with relapse. A comprehensive search was carried out across six major online databases using relevant keywords pertaining to our study, including "open bite relapse," "orthodontic retention," "orthodontic surgery," "orthodontic appliance," "orthodontic management," "orthodontic treatment," "orofacial myofunctional therapy (OMT)," "skeletal anchorage," and "treatment follow-up period." Eleven studies were selected after the application of relevant inclusion and exclusion strategies. The mean follow-up period of treatment for the studies ranged from six months to 4.5 years. Of all the management strategies assessed, OMT was found to be the least effective for OBR management. Surgical management modalities, such as mandibular repositioning and molar intrusion using skeletal anchorage, in conjunction with the usage of orthodontic appliances, were found to be noticeably effective, especially in the cases of participants who were <18 years of age. However, when utilized on a singular basis, either of them was found to be lacking the desired effect. The overall odds ratio (OR) of 0.48 (0.37, 0.64) and risk ratio (RR) of 0.62 (0.51, 0.74) were obtained after the meta-analysis of the different interventions for OBR, indicating statistical significance. There were only 11 studies included in the study, so it's possible that not all management strategies for OBR were fully understood. The limited number of studies may also have affected the generalizability of the findings. Although statistical differences were obtained to a certain degree, more clinical trials are needed to assess the effect of such surgical modalities as a viable management tool for OBR, since these represent a significant limiting factor in terms of the overall cost of the treatment placed upon the patient. Prior to the start of the research, registration was done in accordance with the Preferred Reporting Items for Systematic Reviews and Meta-Analyses (PRISMA) standards. The research protocol was created to meet the goals and was properly filed with the International Prospective Register of Systematic Reviews (PROSPERO) (CRD42023401991).

## Introduction and background

Open bite relapse (OBR) occurs when the vertical gap between the upper and lower front teeth, which had previously been closed or reduced through orthodontic treatment, reverts to its original state or worsens over time. Open bite relapse can happen for various reasons, including factors related to growth and development, changes in oral habits, or inadequate retention measures following initial orthodontic treatment [1].

In orthodontic treatment, the goal is to correct the malocclusion and achieve a stable result that lasts over time [2]. However, achieving long-term stability can be challenging, and many patients experience relapse after treatment, especially in cases of open bite. Relapse occurs when the teeth gradually move back to their original position over time, and this can be a frustrating experience for both the patient and the orthodontist [2]. Several factors can contribute to OBR, including inadequate retention, growth and development, muscle forces, and skeletal changes [2]. Inadequate retention can occur when a patient does not wear their retainer as prescribed, causing the teeth to shift back toward their original position. Growth and development can also play a role, as facial bones can continue to grow and change after orthodontic treatment, leading to relapse. Muscle forces can also impact the stability of the orthodontic result, as the tongue and lip muscles can exert significant forces on the teeth. Skeletal changes, such as changes in the position or angle of the jaws, can also contribute to OBR [3].

There are several management strategies available for preventing and managing OBR. These strategies include retention protocols, orthodontic appliances, and surgical procedures. Retention protocols involve the use of retainers or other appliances to maintain the position of the teeth after orthodontic treatment [3-4]. Different types of retainers are available, including fixed and removable appliances, and the choice of retainer depends on several factors, including the type of malocclusion and the patient's individual needs and preferences [4]. Fixed retainers are bonded to the back of the teeth and are usually recommended for patients with moderate to severe OBR or those who are at high risk of relapse. Removable retainers, on the other hand, can be easily removed by the patient for cleaning and can be used for mild to moderate OBR cases. It is important to note that retention protocols must be followed strictly to prevent relapse [5].

Orthodontic appliances can also be used to manage OBR, including fixed appliances such as braces and clear aligners. These appliances can be used to move the teeth into a more stable position, and they can be customized to fit the individual needs of each patient. Surgical procedures can also be considered in cases of severe OBR, and these procedures can involve repositioning the jaws or altering the shape or size of the teeth [6]. A systematic review of studies that assessed different management strategies for OBR found that the use of retention protocols was the most effective strategy for preventing relapse [7]. The review also found that fixed retainers were more effective than removable retainers, especially in cases of severe OBR. Additionally, the review found that the use of orthodontic appliances such as braces or clear aligners was effective for managing OBR, but the long-term stability of the results was dependent on the patient's compliance with retention protocols [7].

Another systematic review and meta-analysis of studies that assessed the effectiveness of surgical procedures for OBR found that these procedures can be effective for managing severe OBR. However, the review noted that surgical procedures are associated with a higher risk of complications and may not be appropriate for all patients [7]. The use of titanium mini plates or monocortical bone screws for orthodontic intrusion of the back teeth became feasible with the development of skeletal anchorage [4-7]. Skeletal anchorage enables an anti-clockwise rotation of the mandible with the ensuing bite closure by encouraging the intrusion of molars into the bony support. Through orthognathic surgery, these symptoms are thought to be somewhat comparable to those of a maxillary impaction [7].

The choice of management strategy depends on several factors, including the severity of the OBR, the patient's individual needs and preferences, and the orthodontist's clinical judgment. It is important for patients to follow strict retention protocols after orthodontic treatment to prevent relapse, and for orthodontists to closely monitor patients for signs of relapse and adjust treatment as necessary [8]. Hence, we conducted this systematic review to assess different types of OBR management strategies and determine which of them were the most optimal by conducting a meta-analysis and analyzing its findings. 

In the realm of orthodontics, OBR presents a common challenge where the closure achieved during treatment regresses over time, often frustrating both patients and orthodontists. Several factors contribute to this phenomenon, including growth and development, oral habits, and inadequate retention measures post-treatment. The need for effective management strategies to prevent and address OBR is evident.

This systematic review aims to address the dearth of comprehensive analysis regarding the optimal management strategies for OBR. Despite the availability of various treatment modalities, there remains a lack of consensus on the most effective approach. Furthermore, existing literature lacks clarity on contradictory findings, especially concerning appliance efficacy in relapse prevention. The justification for this review stems from the urgency to streamline OBR management protocols, enhance treatment outcomes, and minimize patient dissatisfaction. By synthesizing available evidence through meta-analysis, this review seeks to provide nuanced insights into the comparative effectiveness of retention protocols, orthodontic appliances, and surgical interventions. One notable gap in the current literature pertains to contradictory findings regarding the efficacy of different appliances in controlling OBR. While some studies advocate for fixed retainers, citing their superior stability, others suggest comparable efficacy with removable appliances. This review endeavors to elucidate these disparities, shedding light on the most reliable appliance choice for OBR management. Moreover, this review aims to highlight the critical importance of adherence to retention protocols post-treatment. Understanding the pivotal role of patient compliance in preventing relapse is imperative for orthodontists and patients alike. Through meticulous analysis and synthesis of available evidence, this systematic review seeks to provide evidence-based recommendations for optimizing OBR management strategies. By elucidating the strengths and limitations of various approaches, this review endeavors to guide clinical decision-making, ultimately enhancing treatment outcomes and patient satisfaction in orthodontic practice.

## Review

Materials and methods

Population, Intervention, Control, and Outcomes (PICO) Strategy

We followed the PICO strategy for this study, which was as follows: Population: Individuals with open bite relapse or undergoing treatment for open bite correction; Intervention: Various management strategies are employed for open bite relapse or correction; Comparison: Different management strategies compared to each other or no intervention; and Outcome: Assessing the efficacy of management strategies in preventing or minimizing the recurrence of open bite relapse and mitigating associated complications.

Search Strategy

A comprehensive search strategy was developed and implemented across six different databases concerning this review. The databases utilized were PubMed, the Excerpta Medica database (EMBASE), Web of Science, Scopus, and the Cochrane Library. The search strategy incorporated a combination of Boolean operators (AND, OR) to capture relevant studies. The Medical Subject Headings (MeSH) keywords used in the search strategy included "Open bite," "Relapse," "Management," "Treatment," "Orthodontic retention," "Skeletal anchorage," and "Retention protocol." These MeSH terms were combined using Boolean operators to refine the search results. For example, the search string "(Open bite OR OpenBite) AND Relapse" was employed to capture studies related to open bite relapse. In addition to MeSH terms, free-text keywords were also incorporated to ensure a comprehensive search. Synonyms and related terms for each key concept were used, such as "recurrent" and "treatment." These keywords were combined using Boolean operators to refine the search and increase its sensitivity.

The search strategy was adapted to the specific syntax and requirements of each database, including the appropriate placement of parentheses and quotation marks for grouping search terms and phrases as "(Open bite OR OpenBite) AND Relapse AND Management AND Treatment", "(Orthodontic Retention OR Skeletal Anchorage OR Retention Protocol) AND Open bite", "(Treatment OR Management OR Intervention) AND (Open bite relapse OR Open bite correction)", "(Orthodontic Retention OR Skeletal Anchorage OR Retention Protocol) AND (Prevention OR Efficacy OR Complications)". Specific inclusion and exclusion criteria were established to ensure that the included studies aligned with the research objectives and provided reliable evidence on management strategies for OBR, which are provided below.

Inclusion Criteria

Only clinical studies, including randomized controlled trials (RCTs), were eligible for inclusion. These study designs were chosen to capture a range of evidence on the effectiveness of different management strategies. The studies had to include participants with a confirmed diagnosis of open bite relapse. There were no restrictions on age, gender, or other demographic characteristics to allow for a diverse representation of the population. The studies needed to evaluate specific management strategies for addressing open bite relapse. This encompassed various techniques, appliances, or treatments aimed at correcting the condition. Studies with a comparison group, such as an alternative management strategy or a control group receiving no intervention, were included. This facilitated the comparison of different management strategies and their relative effectiveness. The studies had to report relevant outcomes related to open bite relapse, such as changes in occlusion, relapse rates, and any complications or adverse effects associated with the management strategies.

Exclusion Criteria

Studies that did not involve clinical interventions or focused on non-human subjects, such as in vitro studies or animal experiments; studies that did not specifically address the management strategies for open bite relapse were excluded to maintain the focus on the research question. Studies with incomplete or insufficient data to extract relevant information for analysis were excluded to ensure robust and reliable findings; only studies published in the English language were included to facilitate the review process and data extraction.

Data extraction for this review process included retrieving details about study characteristics, participant characteristics, intervention details, comparison groups, outcome measures, and reported results. Study characteristics encompassed study design, publication year, country, sample size, and study duration. Participant characteristics included age range, gender distribution, and specific inclusion and exclusion criteria. Intervention details focused on techniques, appliances, treatments, and intervention parameters. Comparison groups were identified, and outcome measures encompassed open bite relapse, occlusal changes, patient-reported outcomes, and complications. Reported results included statistical analyses, effect sizes, p-values, and confidence intervals. 

Statistical Protocol

After looking at the variables in the papers, the data were put into the program, and forest plots were made and evaluated to show the odds ratio (OR) and risk ratio (RR) of the effects of the different OBR management methods used in the relevant studies. Based on the methodology used in the particular research, various forest plots were created. A fixed effects model with a 95% confidence interval was used for the meta-analysis.

Results

Risk of Bias Assessment

**Figure 1:**
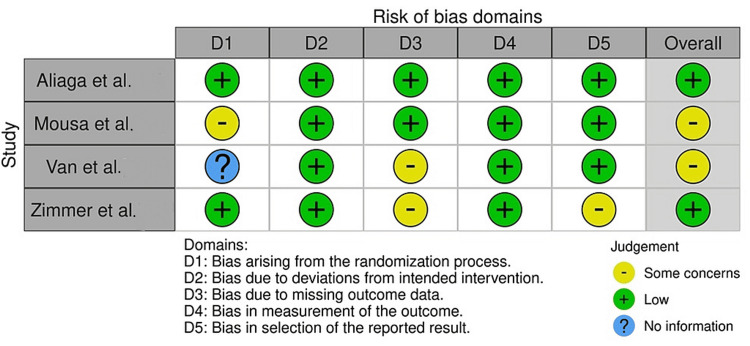
Assessment of the risk of bias in the RCTs selected for the review References: Aliaga et al. [11]; Mousa et al. [14]; Van et al. [20]; Zimmer et al. [21] RCTs: randomized controlled trials

**Figure 2:**
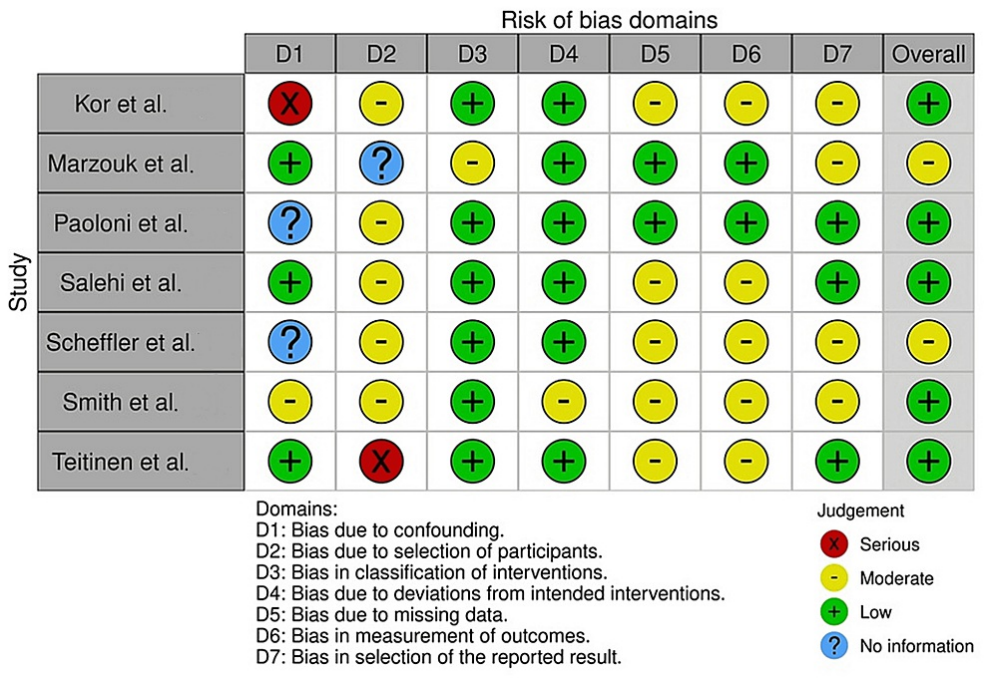
Assessment of the risk of bias in the cohort-based trial selected for the review References: Aliaga et al. [11]; Kor et al. [12]; Marzouk et al. [13]; Mousa et al. [14]; Paoloni et al. [15]; Salehi et al. [16]; Scheffler et al. [17]; Smith et al. [18]; Teitenen et al. [19]; Van et al. [20]; Zimmer et al. [21]

A specific bias assessment strategy was employed to evaluate the methodological quality and risk of bias in the selected clinical studies. Two different tools were used for this purpose: the RoB-2 (Risk of Bias 2) tool for RCTs [9] and the Risk of Bias in Non-randomized Studies of Interventions (ROBINS-I) tool [10], as shown in Figures 1-2 [11-21].

For RCTs, the RoB-2 tool was applied to assess the risk of bias in the following domains: randomization process, deviations from intended interventions, missing outcome data, measurement of the outcomes, and selection of the reported results. Each domain was evaluated using specific signaling questions, and the risk of bias was rated as low, high, or unclear for each domain. The bias assessment was conducted independently by two reviewers, and any discrepancies were resolved through discussion and consensus. For cohort-based and case-control studies, the ROBINS-I tool was utilized to assess the risk of bias in the following domains: bias due to confounding, bias in the selection of participants, bias in the classification of interventions, bias due to deviations from intended interventions, bias due to missing data, bias in the measurement of outcomes, and bias in the selection of the reported results. Similar to the RoB-2 assessment, each domain was evaluated using specific signaling questions, and the risk of bias was rated as low, moderate, serious, or critical for each domain. Again, the bias assessment was conducted by two independent reviewers, with any disagreements resolved through discussion. The use of these bias assessment tools allowed for a comprehensive evaluation of the methodological quality and risk of bias in the included clinical studies. By systematically assessing the risk of bias, the review authors aimed to ensure the validity and reliability of the evidence synthesized in the systematic review and meta-analysis.

Table 1 presents the 11 clinical studies [11-21] focusing on a particular topic, although the specific research objectives and outcomes are not provided.

**Table 1:** Studies selected for the review and their characteristics observed in terms of different variables

Study	Year	Sample size (n)	Mean age (in years)	Gender
Aliaga et al. [11]	2022	50	8.26	31 females
Kor et al. [12]	2014	29	21.68	15 females
Marzouk et al. [13]	2016	26	22.5	15 females
Mousa et al. [14]	2021	40	8.8	19 females
Paoloni et al. [15]	2022	23	9.3	16 females
Salehi et al. [16]	2015	37	18.2	17 females
Scheffler et al. [17]	2014	30	25	19 females
Smith et al. [18]	2010	49	17.8	34 females
Teitinen et al. [19]	2012	24	30.1	
Van et al. [20]	2016	22	7.1-10.6	11 females
Zimmer et al. [21]	2016	129	26.7	-

The sample sizes varied across the studies, ranging from as low as 22 to as high as 129 participants. Regarding the age of the participants, the studies included individuals across a wide range. The mean age reported in the studies varied from 7.1 years to 30.1 years, suggesting that the research covered different age groups or populations. This variation in age indicates the potential inclusion of pediatric, adolescent, and adult populations, each with its own unique considerations and factors affecting the findings. The gender ratio in the studies leaned towards females, with the majority of studies reporting a higher number of female participants compared to males. This gender skew was consistent in all studies except for one, where the gender distribution was not specified. The preponderance of females in the studies could reflect specific population characteristics or the research focus on conditions or issues more prevalent among females.

The findings from the studies presented in Table 2 provide valuable insights into the various correction strategies employed for OBR management.

**Table 2:** Management strategies observed under the selected studies and their inferences observed RCT: randomized controlled trial; OBR: open bite relapse; OMT: orofacial myofunctional therapy

study	Study protocol	Correction strategy employed	Follow-up period assessed	Inference assessed
Aliaga et al. [11]	RCT	Bonded spurs alone and with build-ups	Twelve months after the first treatment	Both treatment protocols were similarly effective for anterior OBR cases.
Kor et al. [12]	RCT	Mandibular orthognathic surgery	One year postoperatively	In the examined groups with little to no OBR, satisfactory occlusal stability was seen at the one-year follow-up.
Marzouk et al. [13]	Cohort	Zygomatic mini plates	Four years after the first treatment	After one year and four years post-treatment, OBR relapsed by 8% and 11%, respectively.
Mousa et al. [14]	RCT	Bionator and posterior bite plane (removable)	12 months after the first treatment	When used early in the therapy of the skeletal anterior OBR, both appliances were successful.
Paoloni et al. [15]	Retrospective	Maxillary Expander, Bite Block, and Quad-Helix Crib	4.2 years after the first treatment	The relapse of early orthodontic treatment in growing individuals with open bites might be linked to the greater depth of the antegonial notch, which is connected to an elongated mandible, as well as an increased gonial angle.
Salehi et al. [16]	Retrospective	Fixed retainers	Three years after the first treatment	Fixed retainers by themselves were ineffective in preventing OBR, and extraction and use of adjunctive removable appliances did not have any effect on the treatment relapse.
Scheffler et al. [17]	Retrospective	Maxillary intrusion splint	Two years after the first treatment	Small changes that occurred more than a year after treatment were mostly due to growth rather than OBR in tooth placements.
Smith et al. [18]	Case-control	OMT	Six years after the first treatment	In comparison to orthodontic therapy alone, OMT was much more effective at maintaining the closure of the anterior OBR.
Teitinen et al. [19]	Retrospective	Maxillary repositioning and mandibular surgery	3.5 years postoperatively	In broad terms, especially when both jaws underwent surgery, the maxilla appeared to relapse considerably vertically and the mandible both vertically and sagittally. When the maxilla had been the only area of surgery, the overbite appeared to be more stable.
Van et al. [20]	RCT	OMT	Six months after the first treatment	OMT was successful in preserving the closure of OBR by influencing tongue behavior.
Zimmer et al. [21]	RCT	Specialised pacifier	27 months after the first treatment	The pacifier used in this study demonstrated slightly more efficacy than the normal pacifier in terms of OBR closure.

The results of an RCT [11] comparing bonded spurs alone and with build-ups showed that both treatment procedures had moderate success rates for anterior OBR correction, with rates of 67% and 72% after a 12-month follow-up. Another retrospective study [12] focused on mandibular orthognathic surgery and found satisfactory occlusal stability at the one-year postoperative assessment in groups with little to no OBR. This suggests that mandibular orthognathic surgery can contribute to stable occlusion in the absence of significant OBR. However, the long-term effectiveness of certain correction strategies raises concerns. A cohort study [13] investigating the use of zygomatic mini plates reported OBR relapse rates of 8% and 11% after one year and four years post-treatment, respectively. Moreover, the study observed high overall OBR and molar incursion relapse rates of 76% and 73% within the first year following treatment. These findings suggest that zygomatic mini plates may not provide long-term stability in OBR correction.

The effectiveness of appliances such as the Bionator and posterior bite planes was examined in an RCT [14]. The study showed initial success when these appliances were used early in the therapy of skeletal anterior OBR. In a retrospective study [15] comparing the outcomes of different correction strategies, including Maxillary Expander, Bite Block, and Quad-Helix Crib, no statistically significant differences were found between the OBR group and the group with no relapse. However, the OBR group had greater values for antegonial notch depth, indicating a potential impact on skeletal changes associated with OBR. Fixed retainers were assessed in a retrospective study [16] and were found to be ineffective in preventing OBR on their own. Cephalometric measures were also unable to reliably anticipate changes in overbite. This suggests that fixed retainers may not provide sufficient stability, and additional measures may be required for effective OBR management. A retrospective study [17] on the use of maxillary intrusion splints found that small changes occurring more than a year after treatment were mostly attributed to natural growth processes rather than OBR-related tooth positioning. This suggests that growth rather than the splint's efficiency in OBR correction was the primary factor influencing changes in tooth placements. In a case-control study [18], the effectiveness of orthodontic therapy alone was compared to the effectiveness of orofacial myofunctional therapy (OMT) in keeping the OBR closed. The study demonstrated that OMT was significantly more effective than orthodontic therapy alone over a six-year period. This highlights the potential benefits of incorporating OMT into OBR management.

Concerning surgical interventions, a study [19] that looked back at maxillary repositioning and mandibular surgery showed that the maxilla dropped down a lot, especially when both jaws were operated on. The mandible showed relapse both vertically and sagittally. However, when the maxilla was the only area of surgery, overbite stability appeared to be more favorable. These findings suggest that combined surgery on both jaws may have a greater impact on relapse compared to isolated maxillary surgery. In an RCT [20] focusing on OMT, the study found that OMT successfully preserved the closure of OBR by influencing tongue behavior at the six-month follow-up. This highlights the effectiveness of OMT in OBR management. Finally, an RCT [21] comparing a specialized pacifier to a normal pacifier for OBR closure demonstrated slightly higher efficacy for the specialized pacifier after a 27-month follow-up. This suggests that the specialized pacifier may have some advantages in terms of OBR closure compared to a regular pacifier.

**Figure 3:**
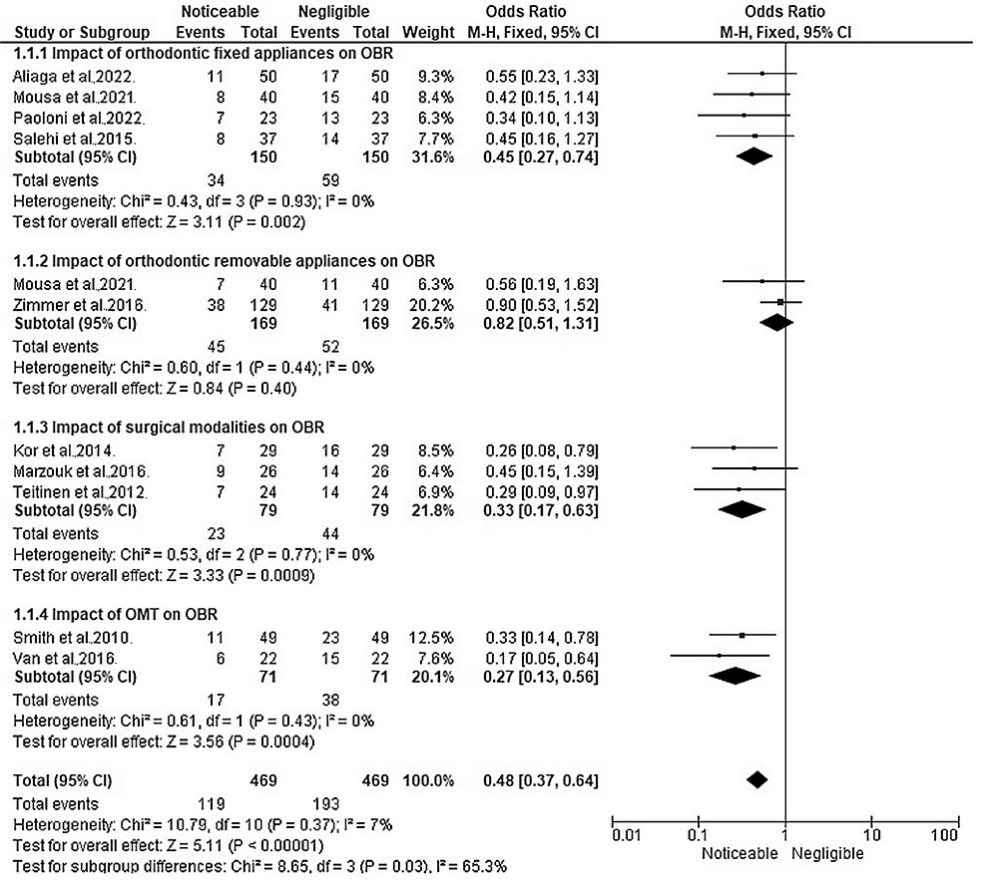
Impact of different management strategies for OBR, represented in terms of OR, on a forest plot References: Aliaga et al. [11]; Kor et al. [12]; Marzouk et al. [13]; Mousa et al. [14]; Paoloni et al. [15]; Salehi et al. [16]; Scheffler et al. [17]; Smith et al. [18]; Teitenen et al. [19]; Van et al. [20]; Zimmer et al. [21] OBR: open bite relapse; OMT: orofacial myofunctional therapy; OR: odds ratio

Figure 3 represents the meta-analysis findings in terms of OR across four different subgroups of interventions that were discussed in this review.

The pooled OR estimate across the first subdivision of this forest plot was 0.45 (95% CI: 0.27, 0.74), indicating a significant association between fixed orthodontic appliances and a reduced likelihood of noticeable OBR as assessed in four studies [11, 14-16]. The forest plot also displayed the heterogeneity test results, which showed no significant heterogeneity among the studies (Chi² = 0.43, df = 3, P = 0.93) and a low I² value of 0%, suggesting minimal inconsistency among the included studies. Additionally, the test for overall effect yielded a significant result (Z = 3.11, P = 0.002), indicating that the combined effect size of the studies was statistically significant. The forest plot visually represented the individual study ORs along with their corresponding confidence intervals, highlighting the consistency of the findings across the studies. The narrow confidence intervals and the significant overall effect support the conclusion that fixed orthodontic appliances have a notable impact on reducing OBR. The pooled OR estimate across the second forest plot of this figure was 0.82 (95% CI: 0.51, 1.31), suggesting no statistically significant association between removable orthodontic appliances and the likelihood of noticeable OBR in the two studies [14, 21] where it was used. The forest plot also presented the results of the heterogeneity test, which revealed no significant heterogeneity among the studies (Chi² = 0.60, df = 1, P = 0.44), with an I² value of 0%, indicating a lack of inconsistency between the included studies. Furthermore, the test for overall effect yielded a non-significant result (Z = 0.84, P = 0.40), indicating that the combined effect size of the studies was not statistically significant. The forest plot visually displayed the individual study ORs along with their corresponding confidence intervals, illustrating the consistency of the findings across the studies. The wide confidence intervals and the non-significant overall effect suggest that there is insufficient evidence to conclude a noticeable impact of removable orthodontic appliances on OBR. The third forest plot of Figure 3 displays the pooled OR estimate to be 0.33 (95% CI: 0.17, 0.63), indicating a statistically significant association between surgical intervention and reduced odds of noticeable OBR in the three studies [12-13, 19] that assessed surgical modality usage. The heterogeneity test indicated no significant heterogeneity among the included studies (Chi² = 0.53, df = 2, P = 0.77), with an I² value of 0%. This implies that the studies exhibited consistency in their effect sizes and did not show substantial variation. Additionally, the test for overall effect yielded a significant result (Z = 3.33, P = 0.0009), indicating a significant overall effect of surgical intervention on OBR. The forest plot results suggest that surgical intervention has a noticeable impact on reducing the odds of OBR, based on the included studies. The OR estimate of 0.33 indicates 67% lower odds of noticeable OBR among individuals who undergo surgical intervention compared to those who do not. The narrow confidence interval (0.17, 0.63) further supports the precision of the effect estimate, indicating a relatively precise estimation of the treatment effect. The final forest plot of Figure 3 shows a pooled OR estimate of 0.27 (95% CI: 0.13, 0.56), indicating a statistically significant association between OMT and a reduced likelihood of noticeable OBR in the two studies [18, 20] where it was utilized. The forest plot visually depicted the individual study ORs along with their corresponding confidence intervals, demonstrating the consistency of the findings across the studies. The heterogeneity test revealed no significant heterogeneity among the included studies (Chi² = 0.61, df = 1, P = 0.43), with an I² value of 0%, suggesting a lack of inconsistency. This implies that the studies are similar in terms of their effect sizes. Furthermore, the test for overall effect yielded a significant result (Z = 3.56, P = 0.0004), indicating that the combined effect size of the studies was statistically significant.

**Figure 4:**
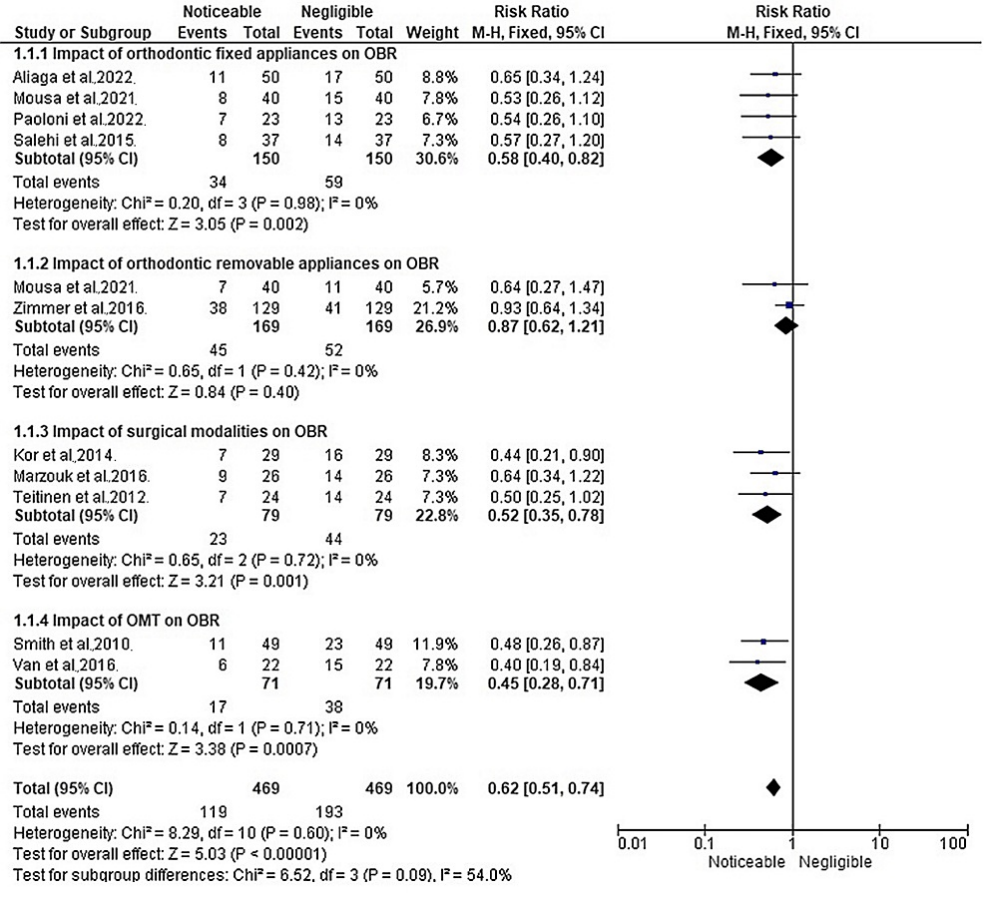
Impact of different management strategies for OBR, represented in terms of RR, on a forest plot References: Aliaga et al. [11]; Kor et al. [12]; Marzouk et al. [13]; Mousa et al. [14]; Paoloni et al. [15]; Salehi et al. [16]; Scheffler et al. [17]; Smith et al. [18]; Teitenen et al. [19]; Van et al. [20]; Zimmer et al. [21] OBR: open bite relapse; OMT: orofacial myofunctional therapy; RR: risk ratio

Figure 4 shows the meta-analysis findings in terms of RR across four different subgroups of interventions that were discussed in this review.

The first subdivision shows the pooled RR estimate was 0.58 (95% CI: 0.40, 0.82), indicating a statistically significant association between the use of fixed orthodontic appliances and a reduced risk of noticeable OBR across the four studies [11, 14-16] that used fixed orthodontic appliances. The forest plot visually represented the individual study RRs and their corresponding confidence intervals, demonstrating the consistency of the findings across the studies. The heterogeneity test indicated no significant heterogeneity among the included studies (Chi² = 0.20, df = 3, P = 0.98), with an I² value of 0%. This implies that the studies were similar in terms of their effect sizes, suggesting a high degree of agreement. Furthermore, the test for overall effect yielded a significant result (Z = 3.05, P = 0.002), indicating that the combined effect size of the studies was statistically significant. The second forest plot in Figure 4, which was based on two studies [14, 21] assessing the usage of removable orthodontic appliances, shows the pooled RR estimate was 0.87 (95% CI: 0.62, 1.21), indicating no statistically significant association between the use of removable orthodontic appliances and the risk of noticeable OBR. The forest plot visually displayed the individual study RRs and their corresponding confidence intervals, providing a comprehensive overview of the results. The heterogeneity test revealed no significant heterogeneity among the included studies (Chi² = 0.65, df = 1, P = 0.42), with an I² value of 0%. This suggests that the studies were consistent in their effect sizes and did not vary significantly. Additionally, the test for overall effect yielded a non-significant result (Z = 0.84, P = 0.40), indicating that there was no significant overall effect of removable orthodontic appliances on OBR. The forest plot results indicate that there is no strong evidence to suggest a noticeable impact of removable orthodontic appliances on OBR based on the included studies. The RR estimate of 0.87 suggests a slightly reduced risk of noticeable OBR, although the wide confidence interval (0.62, 1.21) indicates uncertainty in the effect estimate. It is important to note that the limited number of studies included in the analysis may have influenced the precision and generalizability of the findings. The third forest plot of this figure displays that the pooled RR estimate was found to be 0.52 (95% CI: 0.35, 0.78), indicating a statistically significant association between surgical intervention and a reduced risk of noticeable OBR in the 3 studies [12-13, 19] that utilized surgical interventions. The forest plot visually displayed the individual study RRs and their corresponding confidence intervals, providing a comprehensive overview of the results. The heterogeneity test indicated no significant heterogeneity among the included studies (Chi² = 0.65, df = 2, P = 0.72), with an I² value of 0%. This implies that the studies exhibited consistency in their effect sizes and did not show substantial variation. Additionally, the test for overall effect yielded a significant result (Z = 3.21, P = 0.001), indicating a significant overall effect of surgical intervention on OBR. The forest plot results suggest that surgical intervention has a noticeable impact on reducing the risk of OBR based on the included studies. The RR estimate of 0.52 indicates a 48% lower risk of noticeable OBR among individuals who undergo surgical intervention compared to those who do not. The relatively narrow confidence interval (0.35, 0.78) further supports the precision of the effect estimate, indicating a relatively precise estimation of the treatment effect. The final forest plot of Figure 4 which showed the usage of OMT in two studies [18, 20], displays that the pooled RR estimate was found to be 0.45 (95% CI: 0.28, 0.71), indicating a statistically significant association between OMT and a reduced risk of noticeable OBR. The forest plot visually presented the individual study RRs and their corresponding confidence intervals, providing a comprehensive overview of the results. The heterogeneity test revealed no significant heterogeneity among the included studies (Chi² = 0.14, df = 1, P = 0.71), with an I² value of 0%. This suggests that the studies were consistent in their effect sizes and did not exhibit substantial variation. Additionally, the test for overall effect yielded a significant result (Z = 3.38, P = 0.0007), indicating a significant overall effect of OMT on OBR. The forest plot results indicate that OMT has a noticeable impact on reducing the risk of OBR, based on the included studies. The RR estimate of 0.45 suggests a 55% lower risk of noticeable OBR among individuals receiving OMT compared to those without such therapy. The narrow confidence interval (0.28, 0.71) further supports the precision of the effect estimate, indicating a relatively precise estimation of the treatment effect.

Discussion

The findings from the review of the selected clinical studies and the subsequent meta-analysis provide significant insights and future implications for the study on the impact of different interventions on OBR. The significance of these findings lies in providing evidence-based guidance for clinical practice and treatment decision-making. The results suggest that fixed orthodontic appliances, surgical intervention, and OMT can have a noticeable impact on reducing the risk of OBR. These interventions may offer viable treatment options for patients with OBR. However, it is important to consider the limitations of the study, such as the relatively small number of studies included in the analysis and the potential variations in surgical techniques and patient characteristics across studies. The future implications of this study include the need for further research to validate and expand upon these findings. Larger-scale studies with diverse populations and standardized protocols for interventions would enhance the generalizability and reliability of the results. Long-term follow-up studies could provide insights into the sustainability of the effects and help determine the most effective and durable interventions for managing OBR. Additionally, comparative studies that directly compare different interventions against each other would aid in identifying the most optimal treatment approaches. Overall, these findings contribute to the existing knowledge of OBR management and provide a foundation for future research and clinical decision-making in orthodontics.

Open bite relapse can be a challenging orthodontic problem to manage, but with appropriate management strategies, patients can achieve long-term stability and enjoy a functional, healthy, and aesthetic smile. Orthodontic treatment requires a collaborative effort between the patient and the orthodontist, and it is important for both parties to communicate effectively and work together toward achieving the best possible outcome [7-8]. The study aimed to analyze different management strategies for OBR and identify the most effective approach using a meta-analysis. The significance of this study is that OBR is a significant challenge in orthodontic treatment, and despite the use of different retention strategies, relapse may still occur. Therefore, identifying effective management strategies for OBR is essential to prevent relapse and ensure long-term treatment success.

The study conducted a comprehensive search of major online databases using relevant keywords, and 11 studies were selected after applying inclusion and exclusion criteria. The mean follow-up period for the studies ranged from six months to 4.5 years, which is a significant timeframe for assessing the effectiveness of management strategies.

The findings revealed that OMT was the least effective management strategy for OBR. This finding is significant because OMT is often used as a complementary treatment for orthodontic patients to improve their orofacial muscle function, but this study suggests that it may not be effective in preventing OBR. This finding highlights the need for alternative management strategies for patients who are at risk of OBR [16, 18].

The study also found that surgical treatments like mandibular repositioning and molar intrusion using skeletal anchorage, when used with orthodontic appliances, were very effective, especially in patients younger than 18 years old. This finding is significant because it suggests that surgical management may be a viable option for preventing OBR in younger patients. However, the study also highlights the need for more clinical trials to assess the effectiveness of these surgical modalities, as they can be costly for patients. The observations suggest that using a combination of management strategies may be more effective than using them in isolation. For example, using surgical management modalities in conjunction with orthodontic appliances may be more effective than using either of them alone. This finding is significant because it suggests that a comprehensive treatment approach that combines different management strategies may be necessary to prevent OBR effectively [7, 8, 12, 14, 17].

This investigation's significance lies in its contribution to identifying effective management strategies for OBR. The study's findings suggest that OMT may not be an effective management strategy, while surgical management modalities in conjunction with orthodontic appliances may be more effective, especially in younger patients. However, more clinical trials are needed to assess the long-term effectiveness of these surgical modalities and their overall cost-effectiveness. Overall, the study's findings highlight the need for a comprehensive approach to OBR management that combines different strategies to achieve long-term treatment success.

Skeletal anchorage devices, which require little to no patient cooperation, can be used to execute conventional molar intrusion more effectively [20]. Such a method offers an option for orthognathic surgery [8, 12, 17], which itself may be linked with increased postoperative discomfort, be more expensive, necessitate hospitalization, and require longer postoperative recovery times [8, 21]. The possibility of recurrence following orthognathic surgery is also important [12, 17]. The relapse rate for open bite treatments involving extractions is reported to be 25.8% in earlier studies [22], compared to 38.1% in cases without extractions, despite the fact that there are other orthodontic therapies available. These procedures require a longer course of treatment [23] than those supported by skeletal anchorage treatment, in addition to relapse changes.

According to the literature, using skeletal anchorage devices to carry out this kind of intrusion can cause the mandible to rotate anticlockwise, enhancing facial aesthetics [24-26]. Even though there are a lot of articles that look at the effects of treatment with skeletal anchoring devices, including a systematic review [7], not many studies have looked at the stability of open bite treatment through molar intrusion using skeletal anchorage over the long term. No study has used a randomized strategy to investigate this problem. In order to assess leavers accurately, prospective studies are crucial. This systematic review only included retrospective studies, so relapsed patients may have received new treatment and weren't considered in the analysis of the main studies. As a result, the results of these investigations may significantly underestimate the rate of relapse.

Because maxillary incisor extrusion and molar intrusion are caused by the mandible turning counterclockwise, open bite correction happened in all of the trials. Various major side effects, including first and second molar extrusion [27] and increased overbite [24-26], were seen after the course of treatment. However, in addition to the previously noted effects, it has been noted that after a year of follow-up, there is a minimal vertical recurrence of the maxillary (18%) and mandibular incisors [24, 27].

The results of the meta-analysis revealed some recurrence of mandibular molar encroachment, particularly after the second year of follow-up. Since these values tend to rise over time, it is necessary to employ more successful retention strategies during and after the first year following therapy. In order to reduce the level of relapse described in the literature, it is also important to pay attention to other variables that affect the retention period [24]. The meta-analysis provided a general idea of changes over a brief follow-up period. However, using a meta-analysis may draw criticism due to the acknowledged methodological heterogeneity among the included works. Studies conducted before and after treatment [12] have a sizable bias risk. As a result, the summaries need to be used with care.

However, greater stability was observed for the maxillary molars after 1 year of follow-up, showing a rate of relapse of around 12% [25-27], with values ranging between 13 [27] and 21 [28] in the second year post-treatment. When analyzing the stability of molar intrusion, a greater relapse of 27.2% was observed for the first mandibular molars and 30.3% for the second lower molars [29]. After three years, the post-treatment relapse rate was 18% [29], with 80% of these alterations taking place in the first year following treatment. Overall, these relapse values result in a success rate of 77% after three years of follow-up, which is comparable to what was seen after orthognathic surgery, which varied from 79% after three years [30] to 85% after five years [31, 32].

One year following treatment, Lee et al. [27] found that 18% of patients had an overbite relapse; in contrast, Deguchi et al. [29] and Scheffler et al. [17] recorded relapse rates of 16%-12%, respectively. After four years of therapy, Marzouk et al. [13] found a relapse rate of 11%. According to the literature, there is a 30% relapse for anterior open bite therapy after 10 years of follow-up [33], correlating with the findings of Deguchi et al. [29], who reported the same percent of instability after two years of follow-up.

Regarding skeletal anchorage devices, patients treated with upper mini plates combined with acrylic plates or transpalatal bars had the lowest rate of overbite relapse after a year of follow-up [25-27], whereas patients treated with L-shaped mini plates in the mandibular cortical bone had the highest number of changes [25]. The disparity in bone density between the maxilla and mandible can be used to explain the latter results [34, 35].

The values found in the mandibular rotation anticlockwise tend to decline after the first year of follow-up [25, 29], indicating that the mandible rotates clockwise over time in these samples. Regarding the morphological effects, most of the articles present in the literature demonstrate a reduction in anterior facial height and facial convexity, with only one study being an exception [28]. A reduction in the labial opening and an improvement in the anteroposterior mandibular relationship are other two of these impacts that are mentioned [28, 29].

This study has several limitations that should be considered when interpreting the findings. First, the specific research objectives and outcomes of the 11 included clinical studies were not provided, which makes it difficult to fully understand the scope and focus of the investigations. The variation in sample sizes across the studies, ranging from 22 to 129 participants, indicates differences in the scale and robustness of the research. Additionally, the age range of the participants varied from 7.1 years to 30.1 years, encompassing pediatric, adolescent, and adult populations. This variation in age groups introduces potential confounding factors and limits the generalizability of the findings to specific age demographics. Another limitation is the gender skew in the studies, with the majority of them reporting a higher number of female participants compared to males. This gender imbalance could be due to specific population characteristics or the research focus on conditions more prevalent among females. However, this gender disparity raises questions about the representativeness of the samples and the potential influence of gender on the study outcomes. The study also highlights the long-term effectiveness concerns of certain correction strategies. For example, a study that looked back at the use of zygomatic mini plates found that OBR and molar incursion recurrence rates were high in the first year after treatment. This suggests that this method may not correct OBR in a way that is stable over time. Similarly, the study evaluating fixed retainers found them to be ineffective on their own in preventing OBR, indicating the need for additional measures for effective management. Only the studies published in the English language were included to facilitate the review process and data extraction. These limitations highlight the challenges of achieving lasting results with certain interventions and the importance of exploring alternative or adjunctive approaches. Moreover, the absence of specific details on the research objectives and outcomes of the individual studies makes it challenging to fully evaluate the quality of the evidence and assess potential sources of bias. Further information on study designs, randomization methods, blinding, and control groups would enhance the reliability and validity of the findings.

## Conclusions

This study underlines the importance of a multifaceted approach to OBR management, combining various strategies tailored to the patient's individual needs, age, and specific malocclusion characteristics. The success of orthodontic treatment in preventing relapse is highly dependent on patient compliance, particularly with retention protocols, and the orthodontist’s clinical judgment. Despite the valuable insights, the review acknowledges limitations, including the small number of studies and potential biases within them, which may affect the generalizability of the findings. Moreover, the varying methodologies and follow-up periods across studies necessitate a cautious interpretation of the data.

In conclusion, while fixed orthodontic appliances and surgical interventions show promise in managing OBR, there is a clear need for further clinical trials to explore their long-term effectiveness and cost-efficiency. The potential of OMT as a supportive treatment should also be considered, emphasizing patient-specific treatment plans for optimal outcomes. Continuous monitoring and adaptation of management strategies are vital to mitigate the risk of OBR and ensure stable, long-term orthodontic results. The findings guide clinicians in selecting the most effective treatment protocols and reinforce the importance of personalized care in orthodontics.
